# Mediterranean Diet and Its Association with Cardiovascular Disease Risk Factors: A Scoping Review

**DOI:** 10.3390/ijerph191912762

**Published:** 2022-10-06

**Authors:** Leigh Ann Richardson, Kenneth Izuora, Arpita Basu

**Affiliations:** 1 Department of Epidemiology and Biostatistics, University of Nevada at Las Vegas, Las Vegas, NV 89154, USA; 2 Section of Endocrinology, Department of Internal Medicine, University of Nevada at Las Vegas, Las Vegas, NV 89102, USA; 3 Department of Kinesiology and Nutrition Sciences, School of Integrated Health Sciences, University of Nevada at Las Vegas, Las Vegas, NV 89154, USA

**Keywords:** Mediterranean diet, cardiovascular disease, atherosclerosis, diabetes

## Abstract

Atherosclerosis is the underlying cause of cardiovascular diseases (CVD) and is interrelated to stroke, heart attack, and heart failure. The Mediterranean Diet (MedDiet) has been closely associated with reduced CVD morbidity and mortality, but research is not well explored for this relationship in individuals with diabetes (who experience greater CVD morbidity and mortality than individuals without diabetes). The aim of this review was to explore the literature related to the MedDiet and atherosclerosis and associated risk factors in individuals with and without diabetes. In total, 570 articles were identified, and 36 articles were included. The articles were published between 2011 and 2021. Platforms used for the search were PubMed, Scopus, Cochrane Library, and ProQuest. Our literature search included clinical and observational studies. Clinical studies revealed the MedDiet was associated with improved biomarkers, plaque, and anthropometric measurements that are associated with atherosclerosis and CVD. Observational studies identified associations between the MedDiet and lower presence of atherosclerosis, improved vascular aging, and increased endothelial progenitor cells. However, most of the studies took place in Mediterranean countries. Further research is needed to better understand the long-term effects the MedDiet on atherosclerosis and its associated risk factors in diverse populations to include individuals with and without diabetes.

## 1. Introduction

In the United States, cardiovascular disease (CVD) is the leading cause of death with approximately 655,000 Americans dying each year (Centers for Disease Control and Prevention [1]. CVD is a broad term used to identify many different conditions associated with the cardiovascular system, such as myocardial infarction (MI), heart failure, and stroke. More specifically, atherosclerosis is the major underlying cause of these conditions [2]. Atherosclerosis is described as a chronic form of vascular inflammation, and studies support the close associations between atherosclerosis and diabetes [3,4,5]. This inflammation starts with low-density lipoprotein (LDL)-cholesterol penetrating the arterial intima where it is oxidized, followed by the expression of inflammatory cytokines and chemokines from leukocytes (monocytes and lymphocytes) [6]. Plaque develops when the monocytes differentiate into macrophages and ingest the oxidized LDL, turn into foam cells, which ultimately grow and form plaque [6]. As plaque build-up increases, the arteries become narrower and obstruct blood flow, which can eventually lead to MI, heart failure, stroke, or thrombosis.

Endothelial dysfunction is characterized as a precursor to atherosclerosis. The endothelial cells regulate blood vessel tone and cellular activity, especially the inflammatory pathways for atherosclerosis development [7]. The endothelial cells were long considered a supportive lining with no main function; however, research has identified that endothelial cells play a major role in blood vessel homeostasis [8]. When the endothelium is torn or under a large amount of stress, it can result in a hypercoagulated state and promote blood clotting leading to thrombosis [7,8]. While there are certain unmodifiable factors related to endothelial dysfunction, atherosclerosis, and CVD development (like age), modifiable factors, such as diet and other lifestyles can have a significant impact in reversing or delaying these CVD risks and complications. 

Despite the abundance of research that has identified some of the most common and modifiable risk factors associated with atherosclerosis and CVD development, such as a healthy diet, physical activity, and smoking cessation, the US population continues to struggle with meeting nutritional and physical activity requirements. Diet continues to be the focus of research on cardiovascular health. It is estimated that one in five premature deaths worldwide could be avoided by an optimal, balanced diet [9]. However, an optimal, balanced diet is not as clearly defined. While many dieticians and researchers will agree that increased consumption of fruits and vegetables is required, there are variations in other dietary requirements such as protein types (plant v. animal), grains (whole v. refined), and alcohol consumption, are just a few areas where differences are noted [10,11,12]. However, one diet has consistently been identified on a global scale to promote cardioprotective mechanisms and reduce risk factors associated with atherosclerosis and CVD: the Mediterranean diet (MedDiet).

The MedDiet has been of popular interest in research since its first study in 1958 after Keys identified those in Mediterranean countries had a significantly lower 15-year death rate [13,14]. During Keys research, five cohorts from the Mediterranean shared similar dietary characteristics: 15–20% of total dietary energy was from olive oil, 15–30% from wine, and individuals consumed more (unspecified amount) of fruits and vegetables than people in other areas, such as the Netherlands, Finland, Japan, Yugoslavia, and the U.S. [13]. Since then, the MedDiet has been applied and studied in various countries around the globe and has included participants from an array of backgrounds [15,16,17,18]. Calculating adherence to a MedDiet has varied definitions for assigning points for individuals who meet certain criteria [19]. The complication with defining a single MedDiet is because of the varied populations that live in the area as well as the regional diversity in agriculture [19]. However, the MedDiet is characterized by its high intake of vegetables, legumes, fruits, nuts, and whole grain cereals; primary consumption of olive oil as fats; and low to moderate intake of dairy products, fish and meat, and red wine [20]. In the systematic review by Zaragoza-Martí et al., MedDiet adherence scores were assessed, and many articles measured adherence scores based on the populations. For example, many studies assigned one-point for consuming above the mean of the group for high intake items like fruits and vegetables or when consuming less than the mean for low intake foods like meat [20]. Additional studies assigned one-point for daily consumption (or absence for low intake foods) and others used tertiles to assign scores [20]. 

One of the first MedDiet adherence scores was developed by Trichopoulou et al. [21]. The score ranged from 0–8 points (higher scores equated to higher adherence) and assigned one-point to individuals who consumed at or above the sex-specific medians for vegetables, legumes, fruits and nuts, cereals, as well as a higher monosaturated/saturated fatty acid ratio; one-point was assigned if individuals consumed below the median cut-off for meat and dairy products. For wine, one-point is assigned to men who consume between 10 and 50 g per day and to women who consume between 5 and 25 g a day [19]. In contrast, the Zito et al. MedDiet calculation used specific quantities and frequencies to assign points [22]. In the Zito et al. scoring, one point was assigned if individuals consumed greater than or equal to one serving per day of fruit, vegetables or salads, and if at least one serving of fruit and one serving of vegetables (jointly) were consumed in one day; greater than or equal to one glass of wine; greater than or equal to two servings of legumes; greater than one spoon of olive oil; greater than or equal to three servings of fish; and less than one serving of meat per day; and less than or equal to one serving per day of white bread and less than or equal to one serving per week of rice (or if the individual consumed greater than 5 servings per week of whole-grain bread) [22].

Research has also investigated the role of the individual components of the MedDiet and the relationship between these components and CVD-related diseases and risk factors [23,24]. Red wine and olive oil have been to a greater extent investigated for their individual roles in reducing CVD-related morbidity and mortality [25,26,27,28]. However, the health benefits associated with the MedDiet are predominately due to the overall diet and how the collective dietary components can target multiple, different risk factors [29]. The MedDiet’s holistic approach has been identified with improving risk factors of CVD, especially elevated blood pressure, altered gut microbiome, and elevated blood sugar and lipid profiles [30]. Additionally, research has revealed that the MedDiet may have anti-inflammatory effects on the vascular wall and may modulate expression of pro-atherogenic genes [30]. Individuals with type 2 diabetes (T2D) have been shown to benefit from a MedDiet; however, research is limited for type 1 (T1D) groups [31,32,33]. 

Understanding the association and causality between diet and CVD development is particularly important for individuals with diabetes who are twice as likely to have an MI or stroke than their non-diabetic counterparts [34]. In patients with diabetes, dyslipidemia is promoted by insulin deficiency and/or insulin resistance and is accompanied with increased LDL oxidation, which leads to the development of atherosclerosis [35]. In addition, those with diabetes often have elevated triglyceride levels, which can result in increased production of small, more atherogenic LDL [35]. Because of these challenges, healthy dietary patterns play an important role in the cardiovascular health of individuals with diabetes. The MedDiet has anti-inflammatory and antioxidant compounds that lower inflammation, oxidative stress, and endothelial dysfunction, which are the underlying complications leading to atherosclerosis and CVD [36]. 

Although the relationship between the MedDiet and CVD has been studied in various populations across the world, individuals with T1D are not fully represented in this research and are largely left out of clinical trials that investigate the relationship between a MedDiet and atherosclerosis [4,33,37]. However, individuals with T2D have been included in recent studies, but much of the research focuses on dietary patterns and glycemic control rather than atherosclerosis [30,36]. The MedDiet has been associated with prevention of progression from pre-diabetes mellitus to T2D; identified as a tool for primary prevention of T2D; and improved overall management of T2D and lessened CVD risk [32,38,39]. The MedDiet has also been associated with regressions in Metabolic Syndrome and its individual components, such as BMI and blood pressure [40]. Specifically, patients with T2D who followed a MedDiet experienced improved systolic blood pressure, triglycerides, ratio of total to HDL-cholesterol, and HDL-cholesterol [38]. Recent research studies have found an inverse relationship between higher MedDiet scores and decreased CVD incidence and mortality [41]. However, the authors also acknowledged more clinical research is needed for the association between the MedDiet and potential benefits in only individuals with diabetes [41]. To our knowledge, no other studies have reported a summary on the role of the MedDiet in diabetes patients on atherosclerosis and associated risk factors. 

A second gap in research is the variation in MedDiet and CVD studies with diverse populations. Many of the long-term clinical and observational studies examining the outcomes associated with the MedDiet have been conducted in European countries like Spain and Italy, who are more likely to follow the traditional Mediterranean-styled diet and foods than elsewhere [42]. It is suggested that the MedDiet be assessed for its effectiveness in U.S. populations with special emphasis on reducing elements that contrast with key MedDiet concepts [42]. Lastly, most research in the U.S. and other countries has focused on the MedDiet in primarily white populations and lacks racial/ethnic minority groups [43].

Thus, the current review summarizes the applications of the Mediterranean Diet in various settings and populations with and without diabetes to assess its impact on atherosclerosis development and associated risk factors.

## 2. Materials and Methods

**Figure 1:**
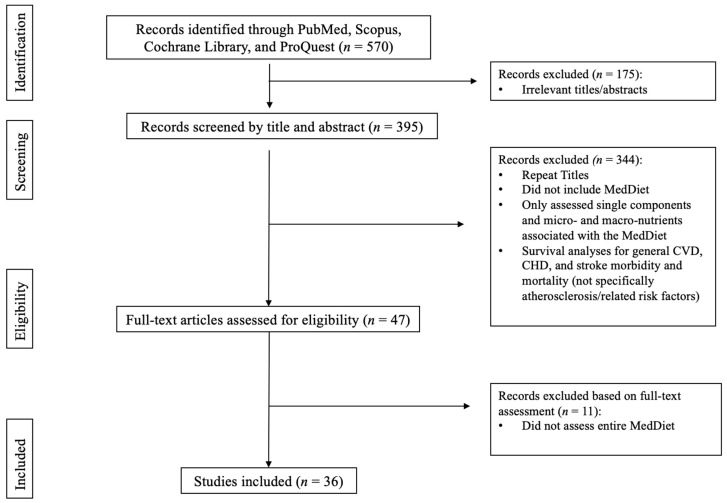
Article Selection.

A literature search was conducted using databases PubMed, Scopus, Cochrane Library, and ProQuest. The search focused on observational and clinical trials applying the MedDiet to various populations (with and without diabetes) and assessing outcomes associated with atherosclerosis, endothelial dysfunction, vascular dysfunction, carotid intima-media and/or inflammatory markers. Selected articles were published from 2011 to 2021. Search terms included “Mediterranean and Atherosclerosis”, “Mediterranean, Atherosclerosis, and Diabetes”, “Mediterranean and Endothelial Dysfunction”, and “Mediterranean, Vascular Dysfunction or Carotid Intima-Media”. Filters were set to full-text, peer-reviewed articles published between 2011 and 2021, in English, and applicable to human adults, (≥18 years-old). With these filters applied, the search produced 648 articles, which were first assessed by their title and abstract, and secondly by MedDiet applications and outcomes related to atherosclerosis, inflammatory markers, artery calcification, and/or plaque presence (Figure 1). Articles whose title or abstract matched these criteria were then reviewed by examining their full text. Ultimately, 36 articles were eligible and included. All authors agreed on the inclusion of the articles. 

Inclusion criteria included: (1) human subjects; (2) adults (≥18 years-old); (3) the application and assessment of a MedDiet; (4) measurement of atherosclerosis, inflammatory markers, artery calcification, and/or plaque presence. Exclusion criteria included: (1) nonhuman models; (2) gestational diabetes; (3) youth or those younger than 18 years-old; (4) omission of a specified MedDiet; (5) omission of data related to artery measurements or inflammatory markers associated with atherosclerosis; (6) only assessing individual (rather than collective) components of the MedDiet. L.A.R. executed the search, reviewed articles for eligibility criteria, and collected the articles for data collection and review. A.B. assisted with the search and confirmed that selected articles met the eligibility criteria.

## 3. Results

Table 1 includes the individual components of the MedDiet and their constituent bioactive compounds that are associated with decreasing cardiovascular disease risk factors. While most compounds have been thoroughly explored in the literature, meta-analyses and systematic reviews of dairy and its cardiometabolic risks have stated more Randomized Control Trials (RCTs) are needed to fully elucidate the benefits and risks associated with dairy and cardiovascular health [44]. For the scoping review results, 17 of the 36 total articles were randomized control trials (RCT) and 19 were observational studies.

### 3.1. Clinical Trials

The RCTs study populations ranged from 20 to 1139 participants with seven studies having less than 100 participants (Table 2). Study durations varied from two hours to 8.1 years with 10 studies lasting a year or longer and the remaining studies were less than four months. Seven studies included individuals who were at high risk of CVD and aged between 55–80 years old in men and 60–80 years old in women. 13 studies included individuals with diabetes. For study locations, 12 studies were based in Spain, three in Italy, one in Poland, and one in Israel. Secondary studies were included in this section if the original studies were RCTs, which lead to many studies utilizing similar cohort populations. 

### 3.2. Genetics

Among the RCTs we reviewed, four studies investigated if a MedDiet was associated with a significant change in genes related to inflammation and plaque stability [51,52,53,54]. The first study compared three dietary patterns over a nine-week period with each diet lasting three-weeks: a MedDiet, saturated fatty acid (SFA)-rich diet, and a low fat, high carbohydrate (CHO-PUFA) diet. The genes studied were associated with inflammation and included nuclear factor kappa B (NF-kB), monocyte chemoattractant protein 1 (MCP-1), tumor necrosis factor (TNF)-α, interleukin (IL)-6, and matrix metalloproteinase 9 (MMP-9) [51]. When compared to the SFA-rich diet, the MedDiet was significantly associated with a decrease in the expression of the p65 subunit of the NF-kB gene and a significant decrease in postprandial expression of the p65 subunit, MCP-1, and MMP-9and an increase in the IkBα subunit of NF-kB gene. When compared to the CHO-PUFA diet, the MedDiet was associated with a significant decrease in p65 and TNF-α expression and an increase in IkBα. 

The second and third study utilized Prevención Con Dieta Mediterránea/Prevention with Mediterranean Diet (PREDIMED) data. In the second study, participants were assigned to a MedDiet with extra virgin olive oil (EVOO) or nuts, or a controlled low-fat diet (LFD) to study the following genes: Toll-like receptor 2 (TLR2), TLR4, TLR6, caspase-1 (CASP-1), inflammasome (NLRP3), IL-receptor 1 (IL1R1), chemokine (C-C motif and C-X-C motif) receptors: CCR2, CCR5, CXCR2 and CXCR3 [52]. The results found no significant differences between the LFD and the two MedDiets for any of the listed genes [54]. For the third study, the MedDiet with nuts was associated with a decline in gene expression of endothelial nitric oxide synthase (eNOS), Caveolin 2 (CAV2), Endothelin (ET)-1 receptors (A and B) and the MedDiet with EVOO was associated with a significant decline in gene expression of CAV2 and ET-Receptor B [53]. 

The final study assessed how different alcohols in combination with either a MedDiet meal or a high fat meal would impact catalase (CAT), superoxide dismutase 2 (SOD2), and glutathione peroxidase 1 (GPX1) expression after two hours of consumption. Overall, the MedDiet meal (alone) was associated with an increase in expression of the three genes, but the authors did not report that these increases were significant. 

### 3.3. Biomarkers for Stress and Inflammation

Thirteen studies included measurements of the MedDiet and its impact on biomarkers for stress and/or inflammation. Six of these studies utilized the PREDIMED cohort results. Two studies with the same first author investigated similar markers from the PREDIMED study and compared baseline data to years 1 and 5, respectively [30,55]. From baseline to year 1 and year 5, there were significant reductions in T-lymphocyte clusters of differentiation (CD)11a, CD49d, CD40 and monocytes CD11a, CD11b, CD49d, and CD40. The authors used different circulatory markers for year 1 and year 5. For circulating marker changes between baseline and year 1, the authors found the MedDiet associated with a decreased in serum vascular cell adhesion molecule (VCAM), serum intercellular adhesion molecule (ICAM), serum E-SEL, serum P-SEL, IL-6, CRP, IL-18, IL-18/IL-10 ratio and a significant increase in TGF-β1. From baseline to year 5, the MedDiet was associated with a significant decrease in MCP-1, IL-6, TNF-α, and hs-CRP. When comparing the MedDiet to the LFD, year 1 showed a significant reduction in monocyte CD40 only, but in year 5 significant reductions were found for all T-lymphocytes and monocytes. For circulatory markers, the MedDiet was associated with a significant decrease in serum P-SEL, IL-6, CRP, and IL-18/IL-10 ratio compared to the LFD. 

Additional PREDIMED secondary analyses included the following biomarkers: hs-CRP, IL-1β, IL1-receptor antagonist (RN), IL-6, IL-8, IL-12p70, IL-18, TNF-α, monocyte chemoattractant protein-1 (MCP-1), regulated upon activation, normal T cell expressed and secreted (RANTES/CCL5), macrophage inflammatory protein (MIP-1β/CCL4), interferon gamma-induced protein 10 (IP-10/CXCL10), and interferon gamma (IFN-γ), epithelial neutrophil-activating protein 78 (ENA78/CXCL5) and inducible T cell alpha chemoattractant (I-TAC/CXCL11), VCAM-1, ICAM-1, vascular endothelial growth factor (VEGF), serum nitric oxide (NO), endothelin-1 (ET-1), serum total antioxidant capacity, serum malondialdehyde (MDA), and Hydroxytyrosol [17,53,54,56]. Overall, findings from these studies found significant reductions in hs-CRP, IL-1β, IL-6, IL-8, IL-12p70, TNF-α, MCP-1, RANTES/CCL5, MIP-1β/CCL4, ENA78/CXCL5, IFN-γ, VCAM-1, ICAM-1, and serum ET-1 following a MedDiet [17,53,54]. Additionally, the MedDiet was associated with significant increase in serum NO [53]. 

One study assessed multiple signaling pathways associated with the MedDiets from the PREDIMED cohort and found MedDiets with either nuts or extra-virgin olive oil reduced hypoxia signaling in the cardiovascular system (CVS), eNOS signaling, nitric oxide signaling in the CVS, renin-angiotensin signaling, aldosterone signaling in epithelial cells, P2 gamma purigenic receptor signaling pathway, and cardiac hypertrophy signaling [56]. Additionally, the MedDiet with EVOO was associated with significant reductions of the following pathways: atherosclerosis, nitric oxide signaling in the CVS, angiopoietin signaling, renin-angiotensin signaling, role of nuclear factor of activated T cells in cardiac hypertrophy, and cardiac hypertrophy signaling [56].

For the six studies not associated with the PREDIMED cohort, one study found a reduction in hs-CRP levels after following the MedDiet and controlled diet (Central European Diet). Additionally, no significant differences between these two diets were identified over time and no significant differences between groups for asymmetrical dimethylarginine (ADMA) were found [57]. A second study discovered the MedDiet was associated with a significant reduction in isoprostane, lipoperoxides, nitrotyrosine, total microparticles (MPs), apoptotic endothelial microparticles (EMPs), activated EMPs, and a significant increase in ischemic reactive hyperemia, beta-Carotene, and percentage of endothelial progenitor cells (EPC) when compared to the two other dietary patterns, which followed a SFA-rich diet or a CHO-PUFA diet [58]. A third study found that compared to a LFD, the MedDiet is associated with decreased EMPs, ROS, cellular apoptosis, senescence and increased cellular proliferation and angiogenesis [58]. This study also found when compared to the LFD, individuals experienced significant increases in percentage of EPCs and significant decreases in EMPs when following a MedDiet [59]. The fourth study found EPC counts for CD34 + KDR+ and CD34 + KDR + CD133+ were significantly higher in individuals who followed a MedDiet compared to a LFD over the course of an 8-year trial [60]. The fifth study identified a MedDiet with EVOO could prevent an increase in ROS-derived NOX2 activation and a reduction in vitamin E serum levels [61]. This study found a MedDiet with EVOO down-regulated NOX2 activation and thwarted serum vitamin E declines following a meal [61]. The final study conducted over a 3-month period in individuals with T2D found a MedDiet was associated with a significant decrease In CRP and ICAM-1 [62]. 

### 3.4. Blood Pressure, Lipids, and Anthropometric Measurements

Eight studies assessed the association between the MedDiet and blood pressure, lipid levels, and anthropometric measurements with four utilizing PREDIMED data. The two related studies with the same first author found the MedDiet was associated with significant reductions in waist circumference (WC) systolic blood pressure (SBP), diastolic BP (DBP), LDL-C, total cholesterol (TC), Total: high-density lipoprotein-cholesterol (HDL-C) from baseline to year 1 and significant reductions in SBP, DBP, LDL-C, TC, Total: HDL-C, triglycerides (TG), weight (wt), body mass index (BMI), and WC from baseline to year 5 [30,55]. When comparing the MedDiet to the LFD, significant reductions were found in SBP, DBP, Total-C, LDL-C, and Total:HDL-C in year 1 data and SBP, DBP, LDL-C, weight, and BMI at year 5. The other two studies found significant reductions in SBP, DBP, LDL-C, BMI, and WC and a significant increase in HDL-C when participants followed a MedDiet compared to a LFD [17,56]. 

The two studies not associated with the PREDIMED cohort found the MedDiet was significantly associated with reductions in oxidized LDL-C when compared to a high fat diet and significant reductions in wt, WC, Glycated hemoglobin (HbA1c), Homeostatic Model Assessment for Insulin Sensitivity (HOMA-IS), TC, TG and SBP when compared to a LFD, which also found a significant increase in HDL-C when following a MedDiet compared to a LFD [52,60]. A third study found no significant changes in BMI, HbA1c, TC, and TG when following a MedDiet for 3 months compared to no intervention [62]. The final study identified a significant decrease in TC, LDL-C, and apolipoprotein B when following a MedDiet compared to a SFA-rich and low-fat, high-carbohydrate diet for 4-weeks each [58].

### 3.5. Carotid Intima-Media Thickness, Flow-Mediated Dilation, and Plaque Height

Six studies investigated the relationship between a MedDiet and arterial health using measurements such as carotid intima-media thickness (cIMT), flow-mediated dilation (FMD), and plaque height. Of the five studies, two utilized data from the PREDIMED cohort, two utilized data from the CORDIOPREV cohort, and two studies were independent [18,59,63,64]. From the two PREDIMED studies, one study found the cIMT decreased in the MedDiet arms when compared to the LFD control group for adjusted models, but this decline in cIMT was only observed when the cIMT was greater than or equal to 0.9 mm [63]. For the second study, researchers identified the MedDiet with nuts was associated with a decrease in the average internal carotid artery (ICA)-IMT, the maximum ICA-IMT, and maximum plaque height [64]. The CORDIOPREV studies found the MedDiet was associated with increased FMD when compared to a LFD; however, another secondary data analysis of the CORDIOPREV data found the MedDiet increased FMD in those with diabetes, but not in those without diabetes [18,59]. The fifth study investigated cIMT regression and progression in newly diagnosed T2D patients [60]. Their research found that individuals who were in the MedDiet arm had higher rates of regression and lower rates of progression for cIMT when compared to those assigned to the LFD arm. The final study also studied individuals with T2D and found a MedDiet compared to no intervention was associated with an increase in FMD percent [62].

### 3.6. MedDiet Adherence

Many of the clinical studies included in this review stem from similar cohorts. The PREDIMED cohort is the most frequently applied cohort and has high adherence to the MedDiet. Overtime, the participants in the cohort increased their MedDiet adherence with mean baseline values around 9 and over years, it has increased to 11 points (out of a 14-point scale). While this is expected for those assigned to the MedDiet groups (EVOO and nuts), individuals assigned to the LFD also saw an increase in MedDiet adherence. Similarly, individuals in the CORDIOPREV cohort also exhibited high adherence to the MedDiet. However, other studies did not report adherence scores in their articles [51,52,57,58,61,62]. 

### 3.7. Observational Studies

Of the 19 observational studies, 15 were cross-sectional, 3 were prospective, and one was retrospective (7-day recall) (Table 3). The three prospective studies had study lengths of 1 year, 5 years, and 9.6 years. Only one prospective study included individuals with diabetes but did not have criteria listed as T1D or T2D, but used clinical measures, such as having blood glucose levels of ≥200 mg/dL, fasting glucose of >126 mg/dL, taking diabetes management medication, or a history of diabetes. Three cross-sectional studies included individual with diabetes with two following similar inclusion criteria of fasting plasma glucose ≥ 126 mg/dL or HbA1c ≥ 6.5% or taking medication for diabetes. The third cross-sectional study included only those with T2D. 

### 3.8. Biomarkers for Stress and Inflammation

Six studies investigated the relationship between biomarkers for stress and inflammation and the MedDiet [15,65,66,67,68,69]. The only prospective study included patients in databases for stroke, transient ischemic attack, and/or atherosclerosis over a one-year period and analyzed the relationship between higher adherence to a MedDiet and the following markers: trimethylamine N-oxide (TMAO), p-cresyl sulfate (PCS), hippuric acid (HA), indoxyl sulfate (IS), p-cresyl glucuronide (PCG), phenyl acetyl glutamine (PAG), and phenyl sulfate (PS). Their results found no significant association between the MedDiet and these biomarkers. 

The remaining five cross-sectional studies investigated the association between the MedDiet and various biomarkers in diverse populations. The first study was conducted in adults who were 20 years old and older with confirmed Familial Hypercholesterolemia or LDL receptor variants and the results found hs-CRP was reduced in those who had the highest MedDiet scores [67]. The second study was conducted in healthy, normal weight adults between 18 and 50 years-old and investigated the association between MedDiet adherence levels (low, medium, and high) and circulating levels of TMAO. The results showed a higher MedDiet adherence was associated with lower levels of TMAO [65]. The next study investigated the association between the MedDiet and EPCs and circulating progenitor cells (CPCs) in a predominately female group of nonagenarians [66]. The results found that a higher adherence to the MedDiet was associated with increased EPCs, but not CPCs. The following two cross sectional studies were conducted in women only and found higher adherence to a MedDiet was not significantly associated with serum VCAM-1, serum ICAM-1, NF-κB and eNOS [68,69]. However, one study did find in young, healthy women a higher adherence to a MedDiet was associated with a reduction in serum E-selectin [69]. 

### 3.9. Insulin Measures, Lipids, and Lipoproteins

Three cross sectional studies investigated the association between a MedDiet and the following insulin measures, lipids, and lipoproteins: very low-density lipoprotein (VLDL), intermediate- density lipoprotein (IDL), apolipoprotein-B (ApoB), LDL-C, HDL-C, TC, TG, TG/HDL ratio, HOMA-IR, Lipoprotein Insulin Resistance (LP-IR) score. From these studies, the MedDiet was associated with reduced levels of ApoB, HOMA-IR, TG/HDL-C ratio [15,16]. However, one study found no significant associations between the exposure and any plasma lipids, lipoprotein particles, or lipoprotein particle sizes [70].

### 3.10. Plaque and Arterial Health

Thirteen studies examined the association between a MedDiet and plaque presence, progression, and overall arterial health. Two studies utilized the ankle brachial index (ABI) to assess peripheral artery disease (PAD) in one group of healthy, 65+ year-olds and in a group of premenopausal women aged 45–54 with asymptomatic CVD. The first study found no association between a MedDiet and ABI; however, the second study did find a significant association between higher MedDiet adherence and normal ABI range [71,72].

For cIMT measurement, two studies found no significant association with MedDiet and cIMT in study populations of adults over age 40 who were stroke free and adults (18+ yo) without diabetes, coronary heart disease or renal failure [16,73]. However, a study that enrolled 150 male patients with clinically stable congestive heart failure found a significant association between the MedDiet and cIMT [74]. 

Other studies examined the association between a MedDiet and plaque in specific locations (femoral, carotid, and iliac, for example), total plaque area (TPA), and plaque thickness. In a study of stroke, transient ischemic attack, and atherosclerotic patients, the MedDiet was not associated with lower TPA [67]; however, in individuals with no stroke history, the MedDiet was associated with a lower TPA and lower plaque thickness [73]. Femoral plaque was inversely associated with higher MedDiet adherence in study populations of men between ages 40–55 and men and women free of clinical CVD between ages 40–54 [75,76]. In the study of men and women between ages 40–54, the MedDiet was associated with lower plaque presence in general and plaque in the aorta, carotids, and iliac [75]. However, the men only study did not find a significant association with plaque in the carotids [76].

The MedDiet was significantly associated with the absence of CAC in three studies of only men between 40 and 55 years old, men and women between ages 40–54 free of clinical CVD, and men and women between ages 45–75 who were free of clinical CHD [75,76,77]. However, one multi-ethnic study of individuals between ages 45–84 did not find a significant association with a MedDiet and absence of CAC [78].

### 3.11. Subclinical Atherosclerosis Measurements

One study of men aged 40–55 and a second study of adults with HIV on antiretroviral therapy for at least 12 months found significant reductions in subclinical atherosclerosis but used different defining criteria. The male only study applied a CAC score of greater than 0 and the presence of plaques in any region (carotid, femoral, and/or iliac) while the adults with HIV study included a cIMT greater than or equal to 0.9 mm and presence of carotid plaques [76,79]. Additionally, in the study group of participants without diabetes, CHD, or renal failure, the researchers included a cIMT greater than or equal to 0.9 mm and presence of carotid plaques but found no significant association in their research [16].

### 3.12. Scoring Measures of Heart and Vascular Health

Four studies utilized scoring measures associated with arterial health and provided information about arterial calcification. The first study found a significant inverse association between the MedDiet and Gensini score in a study population diagnosed with coronary artery disease (CAD) between ages 35–80 [80]. The second study of individuals between 40 and 54 years-old found the MedDiet inversely associated with Agatston score [75]. The third study of adult, men with clinically stable congestive heart found higher adherence to a MedDiet associated with a decreased augmentation index and an increase in the systolic wave of tricuspid annulus [74]. The last study utilized specific criteria to identify early vascular aging (EVA) and normal vascular aging (NVA) and found in adults without chronic conditions, the MedDiet was associated with a decrease in EVA and an increase in NVA [81].

### 3.13. MedDiet Adherence

Overall, adherence to a MedDiet was moderate in most observational studies. Antoniazzi et al. reported 73.5% of the Spanish cohort were categorized in the moderate or strong adherence groups (7–14 points out of a 14-point scale) [15]. However, 83.7% of the Brazil cohort had poor adherence (0–6 points) [15]. Similarly, Gardener et al. found close to 70% of their cohort followed moderate-to-high adherence to a MedDiet [73]. Studies with low adherence were reported by Frölich et al., Gomez Sanchez et al., and the non-HIV group from Viskovic et al. [77,79,81].

## 4. Discussion

From clinical and observational studies, the MedDiet has been associated with beneficial changes in inflammatory gene expression, biomarkers of inflammation and stress, lipids, blood pressure, and anthropometric measures, as well as reduced presence and progression of CAC and other plaque measures. However, more information is needed as studies in this field are predominately from Mediterranean countries and similar cohorts. Additionally, some studies identified a similar decline in a risk factor and others would identify no change. For example, in CAC studies, two studies identified a decreased presence of CAC associated with the MedDiet while two studies did not have this association (versus an increased presence of CAC) [75,76,77,78]. These variations in results are likely due to differences in study design, population characteristics, and the large variations in outcomes. Furthermore, among the reported studies we reviewed, very few studies include individuals with diabetes and CVD risks. Clinical studies were more likely to include individuals with diabetes; however, the studies were limited to similar cohorts and small sample sizes. Observational studies were less likely to include individuals with diabetes and were mostly cross sectional. 

### 4.1. Clinical Studies

As inferred from the clinical studies, a MedDiet has been associated with declines in T-lymphocytes, monocytes, circulating markers (such as CRP, TNF-α, EMPs, and multiple interleukins), blood pressure, LDL-C, and plaque measures. However, these significant reductions were primarily observed in the PREDIMED cohort. Systematic reviews have supported similar findings of MedDiets associated with improved blood pressure and earlier clinical trials have found associations between greater adherence to the MedDiet and increased total antioxidant capacity, decreased oxidized LDL-C, decreased oxidative stress, and reductions in inflammatory and coagulation markers linked with CVD [82,83,84,85]. Researchers suggest these improvements in inflammatory pathways may be due to the high consumption of polyphenols in a MedDiet [86,87,88]. For individuals with T2D, researchers have also suggested the intake of polyphenols is beneficial for those with insulin resistance and T2D risk [87]. However, most authors conclude that more clinical research in diverse populations is still needed to substantiate these findings. 

Major dietary characteristics of the MedDiet include long-chain omega-3 fatty acids from fish and nuts, polyphenols from fruits, vegetables, and wine, probiotics from yogurt and some milk products, and fiber, phytosterols, and antioxidants [89]. Animal models have provided some insights for associations between these dietary components and reduced risk factors associated with atherosclerosis development. Researchers found hypercholesteremic, male rats whose diets were supplemented with omega-3s for 56 days reported an attenuation of an acetylcholine-induced response and improved the vascular function of hypercholesteremic rats to levels similar to normal rates [90]. Ethanol extracts from *Garcinia mangostana L*. peels (contain antioxidant mangosteen) were administered to white mice and were associated with a decrease in LDL through inhibitions of malondialdehyde formation and an increase in HDL cholesterol by preventing transfer of ester cholesterol from HDL to VLDL [91]. Additionally, probiotic-fermented camel milk was associated with a significant reduction in body weight, serum triglycerides, and LDL and an increase in serum HDL in obese, hypercholesterolemic rats [92]. The MedDiet is a holistic diet, and these findings highlight how different components target biomarkers individually, as well as synergistic potential, and collectively change up and down regulation of atherogenic expression. 

Clinical studies included in this scoping review were traditional RCTs with only three following a crossover design. One of the design limitations from these RCTs is study length. 10 of the 17 clinical studies were a year or less. While clinical measures, such as blood pressure, lipids, and biomarkers, exhibit improvements from dietary changes in the short term, longer study periods may provide more information about changes in risk of CVD development especially those related to atherosclerosis and plaque development. Additionally, longer study periods may also provide information about the feasibility of the MedDiet particularly for populations outside of Europe. A second limitation of these studies is the study population size and location. As previously mentioned, eight of the 17 RCT studies were secondary analyses of the PREDIMED cohort. Depending on the research question, the size of the study population ranged from 34 to 1139 participants. More RCTs of diverse populations and larger cohorts are needed to increase power and reliability of the results. Lastly, the inclusion/exclusion criteria favored those who were older and at higher risk of CVD with multiple chronic conditions. 

### 4.2. Observational Studies

Based on the findings from the observational studies, a MedDiet was associated with lower presence of atherosclerosis, better vascular aging, and increased EPCs. While clinical studies provide more evidence of cause-and-effect relationships, observational studies offer information about real life behaviors, but this can introduce more confounding and bias into the study [93]. 

Clinical studies have provided evidence for mechanistic and changes in biological pathways associated with the MedDiet; observational studies have provided information about compliance. In American cohorts, compliance to a MedDiet has been relatively low (25.0 points ± 8.21 for the Framingham cohort and 20.0 points ± 6.5 for Atherosclerosis Risk in Communities (mean ± SD)) [10,94]. In comparison, over the last ten years, Mediterranean countries have reported moderate adherence to a MedDiet with most individuals falling in the moderate adherence category versus many falling into the low and high extremes like American populations [95]. 

Observational studies provide an opportunity to gain information about factors associated with adherence to improve health promotion and consequently health outcomes [95]. In this review, observational studies had more participants who were younger and healthier than clinical study groups, but many excluded individuals with diabetes. Additionally, 14 of the 19 studies included were cross-sectional, which can only provide information about prevalence and associations. To better understand the role of the MedDiet in CVD risk factors, observational studies should include large, diverse cohorts that are studied over longer periods. 

*Clinical v. Observational findings*. Within this review, clinical studies investigated and reported more information on genetics and biomarkers than observational studies. Clinical studies included a more extensive list of diverse biomarkers associated with atherosclerosis development to include TNF-α, VCAM-1, ICAM-1, and several interleukins, which were not as extensively studied in observational studies. Overall, clinical studies reported significant reductions in the listed biomarkers, but in observational studies these were either not studied (no interleukins, TNF-α) or found to have no significant association with MedDiet scoring (VCAM-1 and ICAM-1). Observational studies, however, included more clinical measures of CAC, with most studies including cIMT measurements in their analyses—clinical studies reported more studies with FMD. Overall, clinical studies found the percentage of FMD to increase in individuals who followed a MedDiet and who were also either prediabetic or had T2D. Observational studies findings were mixed for CAC measures, which may be due to the high number of cross-sectional studies. 

### 4.3. Strengths and Limitations

A major strength of this review is the identification of limited information about the applications of a MedDiet and atherosclerosis and its associated risk factors in individuals with and without diabetes. Based on RCTs and observational studies, the data provide a thorough evaluation of the MedDiet in the clinical and real-world situations. This diversity of data highlights differences in outcomes between the two settings. Secondly, the applied search criteria followed a strict set of guidelines. This allowed the current review to discuss the MedDiet in its entirety without focusing on single dietary components. Additionally, the majority of observational studies had large sample sizes and the RCTs included individuals with diabetes. Lastly, an assortment of MedDiet patterns were included to show the diversity in its use and definition. 

This review also experiences some limitations. First, this is a scoping review and does not follow the same rigorous methodology as systematic reviews or meta-analyses. Therefore, we did not conduct any statistical analyses on the data from the various studies and outcomes. The intentions of this review were to highlight the most current research findings that pertain to the application of the MedDiet and atherosclerosis as well as gaps in the research. Secondly, PubMed, Scopus, Cochrane Library, and ProQuest were the only platforms used during the literature search; however, these are major sources for health studies. Additionally, only articles written in English were included. Furthermore, while previously considered a strength, a limitation of the review is the lack of standardized calculation of the MedDiet, which has been a concurrent issue with the variation in application and calculation of the MedDiet in studies. Lastly, the number of observational, longitudinal studies was underwhelming along with limited inclusion of individuals with diabetes. These limitations identify the need for additional research into the potential benefits of a MedDiet over the long-term in individuals with and without diabetes in various global populations. 

### 4.4. Future Directions

Many systematic reviews and meta-analyses have been conducted on the role of a MedDiet and reduced risk of CVD, CHD, stroke, and MI [96,97,98]. Overall, the MedDiet has been associated with declines in these outcomes [96,97,98]. However, there are some key areas for future studies to address on this role of the MedDiet. First, CVD, CHD, stroke, and MI are the clinical manifestations of advanced atherosclerosis. Future studies should assess the MedDiet’s ability to prevent atherosclerosis development, advancement, and if the MedDiet can reduce atherosclerotic plaque over time. Secondly, many studies use T2D as an outcome measure, but for CVD and associated outcomes, individuals with diabetes (T1 and T2) should be included in these studies due to their increased risk of death from CVD. On average, individuals with T1D have had to monitor and live with the disease longer than individuals with T2D [99,100]. Therefore, proven dietary interventions which prevent premature death from CVD and assist in insulin management may be a more feasible option for this at-risk group. Additionally, wine is often contradictory in the literature, but is a component of the MedDiet associated with cardiovascular health benefits. It would be worth exploring how varying ethanol concentrations, frequency, and volume (in the context of the MedDiet) may impact individuals differently based on diabetes and hypertension status and in different racial groups. Lastly, future studies should consider utilizing MedDiet dietary patterns and calculations that measure adherence to a MedDiet while considering food components which do not adhere to a MedDiet, such as the pattern created by Rumawas et al. [10]. This will be particularly important in non-Mediterranean populations as many MedDiet calculations only assess components which align with the MedDiet and do not include those which are not aligned. Once overall adherence to a MedDiet has been established, future studies could investigate dietary substitutions, which may provide similar benefits as the MedDiet, but are more applicable to different geographical locations and populations [101]. 

## 5. Conclusions

The current review is a critical analysis of 36 studies. The aim of this review was to present the most recent clinical and observational study findings pertaining to the MedDiet and atherosclerosis and associated risk factors with a secondary emphasis on inclusion of participants with diabetes. Clinical studies investigating the MedDiet have provided significant results for improved biomarkers, plaque, and anthropometric measurements that are associated with atherosclerosis and CVD. However, these studies are predominately from Spain and Italy as well as similar cohorts. Observational study results were associated with lower presence of atherosclerosis, improved vascular aging, and increased EPCs, but most of these studies were cross sectional and fail to provide more information about long-term dietary habits. Further research is needed to better understand the long-term effects the MedDiet on atherosclerosis and its associated risk factors, and such studies must include individuals with different forms of diabetes, as well as matched controls without diabetes. 

## Figures and Tables

**Table 1:** Individual Components of the Mediterranean Diet and their Constituent Bioactive Compounds and Associations with Cardiovascular Disease (CVD) Risk Factors.

Food Group	Bioactive Compounds	Association with CVD Risk Factors
Fruits	Polyphenols ^3^ Fiber ^2^ Ascorbic acid ^6^ Vitamin C ^6^ Myricetin ^6^ Quercetin ^6^ Anthocyanins ^6^ Cyanidins ^6^ Flavonols ^6^ Ascorbic Acid ^6^	Decrease CVD ^1^ Decrease cholesterol ^6^ Inhibit LDL-C oxidation ^6^ Decrease Blood Pressure ^6^
Vegetables	Polyphenols ^3^ Fiber ^2^ Carotenoids ^6^ Vitamin C ^6^ Lycopene ^6^	Decrease CVD ^1^ Decrease Blood Pressure ^3^ Decrease cholesterol ^6^ Inhibit LDL-C oxidation ^6^
Whole Grains	Phytochemicals ^6^ Fiber ^3^ K^+^, Mg^2+^, and Ca^2+ 3^	Decrease Cholesterol ^3^ Increase Glycemic Control ^3^ Reduce T2DM Risk ^3^ Decrease Blood Pressure ^3^ Decrease Adiposity ^3^
Nuts	Fiber ^3^ K^+^, Mg^2+^, and Ca^2+ 3^ Phytosterols ^6^ Linolenic acids ^2^ Tocopherols ^6^ Omega-3s ^6^	Decrease Cholesterol ^3^ Decrease Triglycerides ^3^ Decrease Blood Pressure ^3^ Reduce T2DM Risk ^3^
Legumes	Polyphenols ^6^ Fiber ^3^ K^+^, Mg^2+^, and Ca^2+ 3^	Decrease CVD ^1^ Decrease Blood Pressure ^3^ Decrease cholesterol ^6^
Olive Oil	Polyphenols ^3^ Phytosterols ^3^ MUFA ^3^	Decrease CVD ^1^ Decrease Adiposity ^3^ Decrease Blood Pressure ^3^ Inhibit LDL-C oxidation ^6^ Decrease Inflammation ^3^ Reduce T2DM Risk ^3^
Fish	Omega-3s ^6^	Decrease Triglycerides ^3^ Increase HDL ^3^ Decrease cholesterol ^6^ Inhibit LDL-C oxidation ^6^ Decrease Blood Pressure ^6^
Red Wine (Moderate)	Polyphenols ^3^ Resveratrol ^2^ Myricetin ^6^ Quercetin ^6^ Anthocyanins ^6^ Cyanidins ^6^ Flavonols ^6^	Decrease Adiposity ^3^ Decrease Blood Pressure ^3^ Decrease Inflammation ^3^ Reduce T2DM Risk ^3^
Meat and meat products ^§^	L-carnitine ^7^ L-carnosine ^7^ Choline ^7^ Lipoic acid ^7^ Conjugated dienes of linoleic acid (CLA) ^7^ Glutathione ^7^ Taurine ^7^ Coenzyme Q10 ^7^ Creatine ^7^	Decrease Blood Pressure ^7^ Decrease Inflammation ^7^ Reduction of Fat Mass ^7^
Dairy	Calcium ^5^ Conjugated linoleic acid ^5^ Glycomacropeptide ^5^ Lactoferrin ^5^ β–Lactoglobulin ^5^	Decrease Blood Pressure ^4^ Decrease Inflammation ^4^ Reduce T2DM Risk ^4^

^§^ Research supports low intakes of lean, unprocessed red meats in a Mediterranean Diet [45]. ^1^ Grosso et al., 2017 [23]; ^2^ Schwingshackl et al., 2019 [39]; ^3^ Salas-Salvadó et al., 2016 [46]; ^4^ Lordan et al., 2018 [47]; ^5^ Majid, 2016 [48]; ^6^ Koutelidakis & Dimou, 2017 [49]; ^7^ Kulczynski, Sidor, & Gramza-Michałowska, 2019 [50].

**Table 2:** Clinical Studies.

Author/Year [Location]	Study Design/Length	Intervention	Participants/Major Cohort	Comparison Group	Diabetes: Y/N	Clinical Measurements	Outcome Associated w/Higher MedDiet Adherence
Medina-Remon et al. (2017) [Spain] [17]	RCT, 1y	MD-EVOO, MD-Nuts, LFD	High CVD Risk, 55–80 yo men & 60–80 yo women (*n* = 1139)/PREDIMED	Baseline Comparison	Y	Urine Total Polyphenols (UTP), SBP, DBP, Fasting Glucose, TC, LDL-C, HDL-C, TG, VCAM-1, ICAM-1, IL-6, TNF-α, MCP-1	UTP T1: ↓UTP, ⦸SBP, ⦸DBP, ⦸Fasting Glucose, ⦸TC, ↓LDL-C, ↑HDL-C, ⦸TG, ⦸VCAM-1, ⦸ICAM-1, ⦸IL-6, ⦸TNF-α, ⦸MCP-1 UTP T2: ↑UTP, ↓SBP, ↓DBP, ⦸Fasting Glucose, ⦸TC, ↓LDL-C, ↑HDL-C, ⦸TG, ⦸VCAM-1, ⦸ICAM-1, ↓IL-6, ↓TNF-α, ⦸MCP-1 UTP T3: ↑UTP, ↓SBP, ↓DBP, ⦸Fasting Glucose, ⦸TC, ↓LDL-C, ↑HDL-C, ⦸TG, ↓VCAM-1, ↓ICAM-1, ↓IL-6, ↓TNF-α, ↓MCP-1
Torres-Pena et al. (2018) [Cordoba, Spain] [18]	RCT, 1.5y	MedDiet v. LFD	CHD Patients w/no events in the last 6 months, 20–75 yo, life expectancy of at least 5 years, prediabetes (*n* = 289), T2D (*n* = 438), and no T2D (*n* = 78) (*n* = 805)/CORDIOPREV	LFD (*n* = 387)	Y	FMD	↑FMD in MedDiet + Prediabetes and MedDiet + Diabetes (Baseline to 1.5y) MedDiet v. LFD in patients with Diabetes: ↑FMD MedDiet & LFD in patients w/o Diabetes: ⦸ FMD
Casas et al. (2018) [Barcelona, Spain] [30]	RCT, 5y	MD-EVOO, MD-Nuts, LFD	High CVD Risk, 55–80 yo men & 60–80 yo women (*n* = 160)/PREDIMED	LFD (*n* = 52)	Y	CVD Risk factors: SBP, DBP, TGs, Total-C, HDL-C, LDL-C, Total:HDL-C, Glucose, Glycated hemoglobin, Wt, BMI, WC T-lymphocytes: CD11a, CD49d, & CD40 Monocytes: CD11a, CD11b, CD49d, & CD40 Circulating Markers: MCP-1, IL-6, TNF-α, & hs-CRP	CVD Risk Factors: Baseline v. 5y: ↓SBP, DBP, LDL-C, Total-C, Total:HDL-C, TG, Wt, BMI, & WC; ↑HDL-C; ⦸Glucose, Glycated Hemoglobin, 5y: MD v. LFD: ↓SBP, DBP, LDL-C, Wt, & BMI ⦸ TG, Total-C, HDL-C, Total-HDL-C, Glucose, Glycated Hemoglobin, & WC T-Lymphocytes: Baseline v. 5y: ↓CD11a, CD49d, & CD40 5y MD v. LFD: ↓CD11a, CD49d, & CD40 Monocytes: Baseline v. 5y: ↓CD11a, CD11b, CD49d, & CD40 5y MD v. LFD: ↓CD11a, CD11b, CD49d, & CD40 Circulating Markers: Baseline v. 5y: ↓MCP-1, IL-6, TNF-α, & hs-CRP 5y MD v. LFD: ↓MCP-1, IL-6, TNF-α, & hs-CRP
Camargo et al. (2012) [Spain] [51]	RCT-Crossover, 9w	MD-VOO, SFA-Rich, CHO-PUFA	Healthy, elderly (*n* = 20)	SFA-Rich and CHO-PUFA	N	Inflammation Genes: NF-kB (p65 & IkBα), MCP-1, TNF-α, IL-6 Plaque Stability Gene: MMP-9 & MIF-1	Compared to SFA-Rich: Fasting: ↓p65, ⦸NF-kB (IkBα), MCP-1, TNF-α, IL-6, MIF-1, MMP-9 Postprandial: ↓p65, MCP-1, MMP-9 ↑IkBα, ⦸TNF-α, IL-6, Compared to CHO-PUFA-Rich: Fasting: ⦸ All measures Postprandial: ↓p65 & TNF-α, ↑IkBα, ⦸MCP-1, IL-6, MMP-9, & MIF-1
Di Renzo et al. (2018) [Rome, Italy] [52]	RCT, 2h	Only: (a) Red Wine (RW), (b) White Wine (WW), (c) Vodka (V); Only: (d) MedDiet or (e) a High-Fat Meal (HFM); Combination: (f)MedDiet + RW (g) MedDiet + WW (h) MedDiet + V (i) HFM + RW (j) HFM + WW (k) HFM + V	18–65 yo, BMI between 18.5–35 kg/m^2^, otherwise healthy adults (*n* = 55)	Baseline Comparison	N	oxLDL-C Gene expression: Catalase (CAT), superoxide dismutase 2 (SOD2), and glutathione peroxidase 1 (GPX1)	Baseline to post-intervention: ↑ oxLDL-C (HFM-only), ⦸ All other beverages, ⦸ All diet, and ⦸All combinations; Among treatments: MedDiet v. HFM: ↓oxLDL-C (MedDiet), ↑oxLDL-C (HFM), MedDiet + RW v. HFM: ↓oxLDL-C (MedDiet + RW), ↑oxLDL-C (HFM) CAT Regulation: ↑RW, ↓WW, V, HFM + WW, & HFM+V SOD2 Regulation: ↑ WW, MedDiet + V, and RW ↓HFM+V GPX1 Regulation: ↑RW, MedDiet+RW, and HFM+RW
Storniolo et al. (2017) [Reus and Barcelona, Spain] [53]	RCT, 1y	MD-EVOO, MD-Nuts, LFD	Non-smoker, hypertensive women, 60–80 yo, not consuming non-steroidal anti-inflammatory drugs w/o CVD, but at high risk of CVD (*n* = 90)/PREDIMED	LFD (*n* = 30)	Y	BP, serum nitric oxide (NO) and endothelin-1 (ET-1) Antioxidant capacities: total antioxidant capacity (TAC), malondialdehyde (MDA) Gene expression: endothelial NO synthase (eNOS), caveolin 2 (CAV2) and endothelin-1 receptors (ETAR and ETBR)	MD-Nuts: ↓DBP, serum ET-1 MD-EVOO: ↑NO; ⦸ antioxidant capacity and MDA Change in Gene Expression: MD-Nuts: ↓eNOS, CAV2, ET-1 receptors (A and B); MD-EVOO: ↓CAV2 and ET-Receptor B
Urpi-Sarda et al. (2021) [Barcelona and Valencia, Spain] [54]	RCT, 3y	MD-EVOO, MD-Nuts, LFD	High CVD Risk, 55–80 yo men & 60–80 yo women (*n* = 285)/PREDIMED	LFD (*n* = 100)	Y	Plasma Markers: CRP, IL-1β, IL-6, IL-8, IL-12p70, IL-18, TNF-α, MCP-1, RANTES/CCL5, MIP-1β/CCL4, IP-10/CXCL10, ENA78/CXCL5, I-TAC/CXCL11 & IFN-γ Genes: TLR2, TLR4, TLR6, NLRP3, CASP-1, IL1R1, CCR2, CCR5, CXCR2, CXCR3	↓ CRP, IL-1β, IL-6, IL-8, IL-12p70, TNF-α, MCP-1, RANTES/CCL5, MIP-1β/CCL4, ENA78/CXCL5, & IFN-γ ⦸ IP-10, I-TAC, & IL-18 ⦸ All Genes
Casas et al. (2016) [Barcelona, Spain] [55]	RCT, 1y	MD-EVOO, MD-Nuts, LFD	High CVD Risk, 55–80 yo men & 60–80 yo women (*n* = 164)/PREDIMED	LFD (*n* = 54)	Y	CVD Risk factors: SBP, DBP, TGs, Total-C, HDL-C, LDL-C, Total:HDL-C, Glucose, Glycated hemoglobin, Wt, BMI, WCT-lymphocytes: CD11a, CD49d, CD40 Monocytes: CD11a, CD11b, CD49d, & CD40 Circulating Markers: sVCAM, sICAM, sE-SEL, sP-SEL, IL-6, CRP, IL-18, IL-10, IL-18/IL-10 Ratio, MMP-9, TIMP-1, MMP-9/TIMP-1 ratio, TGF-β1	CVD Risk Factors: Baseline v. 1y: ↓WC, SBP, DBP, Total-C, LDL-C, & Total:HDL-C; ⦸WT, BMI, Glucose, Glycated Hemoglobin, TG, HDL-C MD v. LFD: ↓SBP, DBP, Total-C, LDL-C, Total:HDL-C; ⦸Wt, BMI, WC, Glucose, Glycated Hemoglobin, TG, & HDL-C T-Lymphocytes: Baseline v. 1y:↓ CD11a, CD49d, CD40 MD v. LFD: ⦸CD11a, CD49d, CD40 Monocytes: Baseline v. 1y: ↓CD40, CD11a, CD11b, & CD49d MD v. LFD: ↓CD40 ⦸CD11a, CD11b, & CD49d Circulating Markers: Baseline v. 1y:↓sVCAM, sICAM, sE-SEL, sP-SEL, IL-6, CRP, IL-18, IL-18/IL-10 ratio; ↑ TGF-β1; ⦸ IL-10, MMP-9, TIMP-1, MMP-9/TIMP-1 ratio MD v. LFD: ↓sP-SEL, IL-6, CRP, IL-18/IL-10 Ratio
Castaner et al., 2013 [Spain] [56]	RCT, 3m	MD-EVOO, MD-Nuts, LFD	men aged 55–80 y, women aged 60–80 y with at least one of the following criteria: (1) T2D or (2) 3 or more CVD risk factors [current smoking, hypertension (BP > 140/90 mm Hg or treatment with antihypertensive drugs), LDL-C concentration > 160 mg/dL (or treatment with hypolipidemic drugs), HDL-Cconcentration < 40 mg/dL, BMI (in kg/m^2^) > 25, or a family history of premature CAD (*n* = 34)/PREDIMED	LFD (*n* = 12)	Y	BMI, WC, SBP, DBP, Glucose, TC, LDL-C, HDL-C, TG, ApoA-I, Apo B-100, OxLDL, CRP, Hydroxytyrosol, IL1β, IL1RN, TNF-α, ICAM1, VEGF Signaling Pathways: Role of NFAT in Cardiac Hypertrophy, P2 gamma Purigenic Receptor Signaling Pathway, Hypoxia Signaling in the CVD, Cardiac Hypertrophy Signaling, Renin-Angiotensin Signaling, Inhibition of Angiogenesis by TSP1, Angiopoietin Signaling, Nitric Oxide Signaling in the CVS, Atherosclerosis Signaling, eNOS Signaling, Factors Promoting Cardiogenesis in Vertebrates, Aldostrone Signaling in Epithelial Cells, Cardiac Beta-adrenergic Signaling, HIF1alpha Signaling, Cardiomyocyte Differentiation via BMP Receptors, Thrombin Signaling, Endothelin-1 signaling, and Cellular Effects of Sildenafil	↓BMI (MD-Nuts & MD-EVOO), ↓WC (MD-Nuts), ↓SBP (MD-VOO), ⦸DBP, ⦸Glucose, ⦸TC, ⦸LDL-C, ⦸HDL-C, TG, ⦸ApoA-I, ⦸Apo B-100, ⦸OxLDL, ⦸CRP, ⦸Hydroxytyrosol Signaling Pathways: (MD-EVOO + MD-Nuts):↓Hypoxia Signaling in the CVS, ↓eNOS Signaling, ↓Nitric Oxide Signaling in the CVS, ↓Renin-Angiotensin Signaling, ↓Aldosterone Signaling in Epithelial Cells, ↓P2 gamma Purigenic Receptor Signaling Pathway, ↓Cardiac Hypertrophy Signaling MD-EVOO (only): ↓Atherosclerosis, ↓Nitric Oxide Signaling in the CVS, ↓Angiopoietin Signaling, ↓Renin-Angiotensin Signaling, ↓Role of NFAT in Cardiac Hypertrophy, and ↓Cardiac Hypertrophy Signaling
Duś-Zuchowska et al. (2018) [57] [Poland]	RCT, 16w	MedDiet v. Central European Diet (CED)	Obese, postmenopausal women, nonsmokers with a risk of Metabolic Syndrome (MS) (*n* = 144)	Baseline Comparison	N	hs-CRP and asymmetrical dimethylarginine (ADMA)	Within group comparisons: ↓hs-CRP Between Group Comparison: ⦸hs-CRP Within group comparisons: ↓ADMA (CED-only) Between Group Comparison: ⦸ADMA
Marin et al. (2011) [58] [Spain]	RCT, Crossover, 4w	saturated fatty acid (SFA) diet; a LFHC diet; and a MedDiet	Free living, elderly (>65 yo), free of chronic illness (hepatic, renal, thyroid, or cardiac dysfunction) (*n* = 20)	Cross comparison	Y	TC, TG, HDL-C, LDL-C, ApoA-I, ApoB, ischemic reactive hyperemia (IRH), Superoxide dismutase activity, β-Carotene, Catalase activity, Isoprostane, Lipoperoxides, ⍺-Tocopherol, OxLDL, Nitric Oxide, Protein Carbonyl activity, Nitrotyrosine, Glutathione peroxidase activity Total MPs, Apoptotic EMPs, Activated EMPs, %EPCs	↓TC, LDL-C, ApoB, Superoxide dismutase activity, Isoprostane, Lipoperoxides, and Nitrotyrosine ↑IRH, β-Carotene ⦸ TG, HDL-C, ApoA-I, Catalase Activity, ⍺-Tocopherol, OxLDL, Nitric Oxide, Protein Carbonyl activity ↓ Total MPs, Apoptotic EMPs, Activated EMPs, ↑ %EPCs
Yubero-Serrano et al. (2020) [Cordoba, Spain] [59]	RCT, 1y	MedDiet v. LFD	CHD Patients w. no events in the last 6 months, 20–75 yo, life expectancy of at least 5 years (*n* = 805)/CORDIOPREV	LFD (*n* = 387)	Y	Flow-Mediated Dilation (FMD), endothelial microparticles (EMPs), and endothelial progenitor cells (EPCs)	When FMD <2% and FMD>2%: MedDiet v. LFD: ↑FMD, ↓EMP (Activated & Apoptotic), and ↑EPC MedDiet v. LFD: ⦸BMI, ⦸LDL-C, ↑HDL-C, ⦸TC, ⦸TG, ↓Fasting Glucose, ⦸Fasting Insulin, ⦸HbA1c, ↓hsCRP ↓EMPs, ROS, cellular apoptosis, senescence ↑ cellular proliferation and angiogenesis
Maiorino et al. (2016) [Naples, Italy] [60]	RCT, 8.1y	MedDiet v. LFD	Men and women with newly diagnosed T2D, overweight, never treated with antihyperglycemic drugs, HbA1c levels <11% (*n* = 215)/MEDITA	LFD (*n* = 107)	Y	endothelial progenitor cells (EPCs) and cIMTWeight, WC, HbA1c, Plasma Glucose, HOMA of insulin sensitivity, TC, HDL-C, Non-HDL-C, SBP, DBP, cIMT, CRP	MedDiet (Baseline, Year 2, Year 4, EOT) and compared to LFD(starting at Year 2): ↑CD34+KDR+, ↑CD34+KDR+CD133+ MedDiet: ↓cIMT; LFD: ⦸cIMT MedDiet (v. LFD): ↓Weight, WC, HbA1c, HOMA-IS, TC, SBP, ↑HDL-C
Carnevale et al. (2014) [Italy] [61]	RCT, Crossover, 4w	Study 1: MedDiet w/EVOO and MedDiet w/o EVOO Study 2: MedDiet w/EVOO and MedDiet w/Corn Oil	Healthy adults working in research institute (*n* = 25)	Cross comparison	N	Platelet reactive oxidant species (ROS) and 8-iso-PGF2a-III, activity of NOX2, the catalytic sub-unit of NADPH oxidase, as assessed in platelets and serum, serum vitamin E and endothelial dysfunction	Study 1: MedDiet w/o EVOO: ↑platelet ROS, 8-iso-PGF2a-III, NOX2 activity, sE-selectin, sVCAM1 and ↓serum vitamin E; ⦸ in all measures for MedDiet + EVOO Study 2: Corn Oil: ↑platelet ROS, 8-iso-PGF2a-III, NOX2 activity, sE-selectin, sVCAM1 and ↓serum vitamin E; ⦸ in all measures for MedDiet + EVOO
Gudban et al. (2021) [Israel] [62]	RCT, 3m	MedDiet Intervention v. No intervention	T2D patients, 18yo+, adults, otherwise healthy (no other chronic diseases or conditions) (*n* = 22)	No intervention (*n* = 10)	Y	BMI, FMD%, CRP, ICAM-1, TC, TG, and HbA1c	⦸ BMI, HbA1c, TC, TG ↓ CRP, ICAM-1 ↑FMD%
Murie-Fernandez et al. (2011) [Navarra, Spain] [63]	RCT, 1y	MD-EVOO, MD-Nuts, LFD	High CVD Risk, 55–80 yo men & 60–80 yo women (*n* = 187)/PREDIMED	LFD (*n* = 62)	Y	Carotid intima-media thickness (cIMT)	1-year change: ↓cIMT in MD+Nuts, ⦸ MD+EVOO, ⦸LFDAdjusted 1 year change: ↓cIMT in MD+Nuts and ↓MD+EVOO, ⦸LFD Univariate & Multivariate analysis: When cIMT >= 0.9 mm, MD-EVOO + MD-Nuts: ↓cIMT, ⦸LFD; For Multivariate Analysis: When cIMT >= 0.9 mm, MD-EVOO ↓cIMT and MD-Nuts ↓cIMT, ⦸LFD When cIMT < 0.9 mm, ⦸All diets
Sala-Vila et al. (2014) [Spain] [64]	RCT, 2.4y	MD-EVOO, MD-Nuts, LFD	High CVD Risk, 55–80 yo men & 60–80 yo women (*n* = 164)/PREDIMED	LFD (*n* = 61)	Y	plaque height and cIMT of three prespecified segments (ICA, bifurcation (BIF), and common (CCA))	LFD: ↑ICA-IMT(Mean), ↑ plaque (max); MD-Nuts: ↓ICA-IMT (mean) ⦸ ICA-IMT (Max) and Plaque (Max); MD-EVOO: ⦸ICA-IMT (Mean), ICA-IMT (Max), and Plaque (MAX) ⦸ Between Group Differences: CCA-IMT (Max and Mean), BIF-IMT (Max and Mean)

⦸ indicates no significant change in outcome when adhering to a MedDiet (or the highest MedDiet score) ↓indicates significant decrease in outcome when adhering to a MedDiet (or the highest MedDiet score ↑indicates significant increase in outcome when adhering to a MedDiet (or the highest MedDiet score). Acronyms: ADMA: asymmetrical dimethylarginine, Apo: Apolipoprotein, BIF: bifurcation (of carotid artery), BMI: Body mass index, BP: Blood Pressure, CAD: Coronary Artery Disease, CASP-1: Caspase 1, CAT: Catalase, CAV: caveolin, CCA: Common carotid artery, CCL: C-C Motif chemokine ligand, CCR: C-C Motif chemokine receptor, CD: clusters of differentiation, CED: Central European Diet, CHD: coronary heart disease, CHO-PUFA: low fat, high carbohydrate (poly-unsaturated fatty acids), cIMT: Carotid Intima-Media Thickness, CRP: C-reactive protein, CVD: Cardiovascular disease, CXCL: C-C Motif chemokine ligand, CXCR: C-X-C Motif chemokine receptor, DBP: Diastolic blood pressure, E-SEL: E-Selectin, EMP: Endothelial microparticles, eNOS: endothelial NO synthase, EPC: Endothelial progenitor cells, ET_R: endothelin-1 receptors, ET: endothelin-1, FMD: Flow-mediated dilation, GPX: glutathione peroxidase, HDL-C: High density lipoprotein, HFM: High fat meal, I-TAC: inducible T cell alpha chemoattractant, ICA: Internal carotid artery, ICAM: Intercellular adhesion molecule, IFN: Interferon, IL: Interleukin, IL1R1: Interleukin 1 receptor type 1, iso-PGF: isoprostane prostaglandin F, LFD: Low-fat diet, MCP: monocyte chemoattractant protein, MD-EVOO: Mediterranean diet—extra virgin olive oil, MD-Nuts: Mediterranean diet—nuts, MDA: malondialdehyde, MIP: macrophage inflammatory protein, MMP: matrix metalloproteinase, MS: Metabolic syndrome, NADPH: nicotinamide adenine dinucleotide phosphate, NF-kB: nuclear factor kappa B, NLRP3: NACHT, LRR and PYD domains-containing protein 3, NO: Nitric oxide, NOX: NADPH oxidase, oxLDL-C: Oxidized low-density lipoprotein, P-SEL: P-Selectin, PREDIMED: Prevención con Dieta Mediterránea, RANTES: regulated upon activation, normal T cell expressed and secreted, RCT: Randomized control trial, ROS: Reactive oxidant species, RW: Red wine, s-indicates serum levels, SBP: Systolic blood pressure, SFA-Rich: Saturated Fatty Acid-Rich, SFA: Saturated Fatty Acid, SOD2: superoxide dismutase 2, T2D: Type 2 Diabetes, TAC: total antioxidant capacity, TC: Total Cholesterol, TG: Triglycerides, TGF: Transforming growth factor, TIMP-1: tissue inhibitor of metalloproteinases, TLR: Toll-like receptors, TNF: tumor necrosis factor, UTP: Urine Total Polyphenols, V: Vodka, VCAM: vascular cell adhesion molecule, VEGF: vascular endothelial growth factor, WC: Waist circumference, Wt: Weight, WW: White Wine.

**Table 3:** Observational Studies.

Authors, Year [Location]	Study Design/Length	Participants	Diabetes: Y/N	Clinical Measurements	Outcome Associated w/Higher MedDiet Adherence
Antoniazzi et al. (2021) [Brazil and Spain] [15]	Cross section	Confirmed Familial Hypercholesterolemia or LDL receptor variants, ≥20 yo (*n* = 190)	N	plasma LDL-C, apolipoprotein-B (ApoB), high sensitivity C-reactive protein (hs-CRP)	⦸ LDL-C, ↓ApoB, ↓hs-CRP
Buscemi et al. (2013) [Italy] [16]	Cross section	No DM, CHD, or Renal Failure, ≥18 yo (*n* = 929)	N	cIMT, HOMA-IR, Triglycerides (Tri)/HDL-C, asymptomatic carotid atherosclerosis (PC)	⦸ cIMT, ↓HOMA-IR, ↓Tri/HDL-C, ⦸ asymptomatic carotid atherosclerosis (plaques and/or cIMT ≥ 0.9 mm)
Barrea et al. (2019) [Italy] [65]	Cross section	Healthy, normal weight, 18–50 yo (*n* = 302)	N	TMAO	↓TMAO
Cesari et al. (2018) [Italy] [66]	Cross section	>90 yo, men and women in Mugello area, Tuscany, Italy (*n* = 421)	N	endothelial progenitor (EPCs) and circulating progenitor (CPCs) cells	4th MedDiet Quartile v. Other Quartiles: ↑ EPCs (CD34+/KDR+, CD133+/KDR+, CD34+/CD133+/KDR+) ⦸ CPCs (CD34+, CD133+, CD34+/CD133+)
Pignanelli et al. (2018) [Ontario, Canada] [67]	Prospective, 1y	Stroke, transient ischemic attack, and/or atherosclerotic patients (*n* = 276)	N	Total plaque area (TPA), trimethylamine N-oxide (TMAO), p-cresyl sulfate (PCS), hippuric acid (HA), indoxyl sulfate (IS), p-cresyl glucuronide (PCG), phenyl acetyl glutamine (PAG), & phenyl sulfate (PS)	⦸ TPA, TMAO, PCS, HA, IS, PCG, PAG, & PS
Shah et al. (2020) [USA] [68]	Cross section	Women enrolled in American Heart Association Go Red for Women Strategically Focused Research Network at Columbia University Irving Medical Center, BMI 25–33 kg/m^2^ or BMI 20–25 kg/m^2^ w/o immediate fam history of obesity, hypertension, or DM; absence of chronic diseases and chornic disease medication (*n* = 25)	N	NF- κB and eNOS	⦸NF- κB and eNOS
Witkowska & Zujko (2014) [Poland] [69]	Cross section	19–22 yo, women, BMI ≤ 25 kg/m^2^, without inflammatory, autoimmune, or metabolic diseases (*n* = 25)	N	sICAM-1, sVCAM-1, and E-selectins	⦸ VCAM-1 and ICAM-1 ↓sE-selectin
Millar et al. (2021) [Ireland] [70]	Cross section	Clinical random sample, 46–73 yo, White European (*n* = 1862)	Y	Plasma Lipids: TotChol, TG, LDL-C, HDL-C Lipoprotein particle concentrations: TotTRL, S-, M-,&L-VLDL; S-, L- & T-LDL; S-, M-, L-, T-HDL; IDL Lipoprotein particle size: VLDL, LDL, HDL, LP-IR	⦸ All Plasma Lipids ⦸ Lipoprotein Particle Concentrations ⦸Lipoprotein particle size
Mattioli et al. (2017) [Italy] [71]	Retrospective, 7d	Asymptomatic for CVD, premenopausal women, 45–54 yo (*n* = 425)	N	ABI	↑ABI (normal range)
Woo et al. (2018) [China] [72]	Cross section	Healthy, ≥65 yo (*n* = 4000)	N	ABI (ankle brachial index)	⦸ ABI
Gardener et al. (2014) [USA] [73]	Cross section	No stroke history, resided in Northern Manhattan, >40 yo (*n* = 1374)	N	cIMT, Plaque Presence, Plaque Thickness, and Total Plaque Area (TPA)	⦸ cIMT, ⦸ Plaque Presence, ↓Plaque Thickness, ↓TPA
Angelis et al. (2020) [Athens, Greece] [74]	Cross section	Clinically stable congestive heart failure (CHF) Males, 18 yo+, left ventricular ejection fraction less than or equal to 40%, symptoms according to New York Heart Association class II or higher, and taking medication (*n* = 150)	Y	PWV: Pulse Wave Velocity; AIx: Augmentation index; cIMT: Carotid Intima Media Thickness; EF: Ejection Fraction; SRV: Systolic Wave of Tricuspid Annulus; LA: Left atrium; GLPS: global longitudinal strain of the left ventricle, VO2 Max, VE/VCO2, Pulse Pressure	↓cIMT, AI x ↑SRV ⦸all other variables
Peñalvo et al. (2016) [Spain] [75]	Cross section	Free of clinical CVD, 40–54 yo (*n* = 4082) [Progression of Early Subclinical Atherosclerosis]	N	Agatston Score (AG), CAC, Any Plaque Presence, Plaque in Aorta (PA), Carotids (PC), Femorals (PF), & Iliac (PI)	(When compared to Western and Social-Business DP):↓AG, ↓CAC Presence, ↓Any Plaque, ↓PA, PC, PF, & PI
Uzhova et al. (2018) [Madrid, Spain] [76]	Cross section	Men, 40–55 yo (*n* = 1798)	N	CACS, Plaque in Femorals (PF) and/or Carotids (PC), and Atherosclerosis	↓PF, ⦸CACS, ⦸PC, ↓Atherosclerosis
Frölich et al. (2017) [Germany] [77]	Prospective, 5y	Free of Clinical CHD, 45–75 yo (*n* = 3718)	Y	CAC	↓CAC progression & CAC degree
Whelton et al. (2015) [USA] [78]	Prospective, 9.6y	Free of clinical CVD, Multi-ethnic, 45–84 yo (*n* = 1850)	N	CAC	⦸ CAC
Viskovic et al. (2013) [Zagreb, Croatia] [79]	Cross section	HIV-infected on ART for 12-months or more, and non-HIV infected, adults 18+ yo, no other serious health conditions or medications (*n* = 241)	N	Subclinical atherosclerosis (defined as CIMT ≥ 0.9 mm and/or the presence of ≥1 carotid plaque)	HIV-Infected: ↓subclinical atherosclerosis (cIMT ≥ 0.9 mm or carotid plaques)
Akgüllü et al. (2015) [Turkey] [80]	Cross section	CAD Diagnosis, 35–80 yo (*n* = 200)	N	GS (Gensini Score)	↓GS
Gomez Sanchez et al. (2020) [Spain] [81]	Cross section	35–75 yo, without CVD or any other chronic conditions (*n* = 500)	Y	early vascular aging (vascular damage in carotid arteries or peripheral artery disease were classified as EVA and subjects at the percentile of the combined Vascular Aging Index (VAI) were classified; ≥p90 was considered EVA and <p90 was considered normal vascular aging (NVA)), carotid-femoral pulse wave velocity (cfPWV), and cIMT	↑MedDiet: ↓EVA ↑NVA

⦸ indicates no significant change in outcome when adhering to a MedDiet (or the highest MedDiet score) ↓indicates significant decrease in outcome when adhering to a MedDiet (or the highest MedDiet score ↑indicates significant increase in outcome when adhering to a MedDiet (or the highest MedDiet score). Acronyms: ABI: Ankle brachial index, AG: Agatston Score, AHA: American Heart Association, AIx: Augmentation index, Apo: apolipoprotein, ART: antiretroviral therapy, BMI: Body mass index, CAC: Coronary Artery Calcification, CACS: CAC score, CAD: Coronary artery disease, CD: clusters of differentiation, cfPWV: carotid-femoral pulse wave velocity, CHD: Coronary heart disease, CHF: Congestive heart failure, cIMT: Carotid intima media thickness, CORDIOPREV: Coronary Diet Intervention With Olive Oil and Cardiovascular Prevention, CPC: circulating progenitor cells, CVD: Cardiovascular disease, DM: Diabetes mellitus, EF: Ejection Fraction, eNOS: endothelial NO synthase, EPC: endothelial progenitor, EVA: Early vascular aging, GLPS: global longitudinal strain of the left ventricle, GS: Gensini Score, HA: hippuric acid, HDL-C: High density-lipoprotein, HIV: human immunodeficiency virus, HOMA-IR,, Hs-CRP: high sensitivity C-reactive protein (hs-CRP), ICAM: Intercellular adhesion molecule, IDL: Intermediate density lipoprotein, IS: indoxyl sulfate, KDR: Kinase insert domain receptor, L-Large, LA: Left atrium, LDL: Low-density lipoprotein, LP-IR: Lipoprotein Insulin Resistance, M-Medium, MEDITA: Methoxyflurane in Emergency Department in ITAly, NF-κB: nuclear factor kappa B, NVA: normal vascular aging, PA: Plaque in Aorta, PAG: phenyl acetyl glutamine, PC: Plaque in Carotids, PCG: p-cresyl glucuronide, PCS: p-cresyl sulfate, PF: Plaque in Femorals, PI: Plaque in Iliac, PS: phenyl sulfate, PWV: Pulse Wave Velocity, S-Small, SRV: Systolic Wave of Tricuspid Annulus, T-Total, TC: Total Cholesterol, TG: Triglycerides, TMAO: trimethylamine N-oxide, TPA: Total Plaque Area, VAI: Vascular Aging Index, VCAM: vascular cell adhesion molecule, VE/VCO2: ventilation/volume of exhaled carbon dioxide, VLDL: Very low-density lipoprotein, VO2: volume of oxygen.
